# Supplementary material for: A hybrid feature extraction framework combining PCA and mutual information for gene expression based lung cancer classification
Source: PLoS One. 2026 Feb 5;21(2):e0342160. doi: 10.1371/journal.pone.0342160 (PMC12875479; doi:10.1371/journal.pone.0342160)
Supplement: Attachment — Submitted filename: Response to Reviewers.pdf [file pone.0342160.s002.pdf]

Response to the Reviewers

We are grateful to the Editor and the reviewers for their thorough review of our manuscript and for their thoughtful and constructive feedback. We have carefully addressed all comments and revised the manuscript accordingly. The changes can be found in the revised manuscript along with this detailed response Letter.

Additional Editor Comments:

Please refine the methods section. Currently, the corresponding section is too long.

Response: Dear Editor, Thank you for this comment. The Methods section has been carefully revised and condensed by removing redundant descriptions while retaining all information necessary for reproducibility.

Please split the results and discussion into two sperate sections, and in the discussion section the current findings, the strengths and limitations of this study should be provided in detail.

Response: Dear Editor, thank you for reading our manuscript carefully, The Results and Discussion have now been separated into two distinct sections. The Discussion section has been substantially expanded to clearly interpret the current findings.

Reviewers' comments:

Reviewer's Responses to Questions

Comments to the Author

1. Is the manuscript technically sound, and do the data support the conclusions?

The manuscript must describe a technically sound piece of scientific research with data that supports the conclusions. Experiments must have been conducted rigorously, with appropriate controls, replication, and sample sizes. The conclusions must be drawn appropriately based on the data presented.

Reviewer #1: Yes

Response: Dear reviewer thanks for the Acknowledgement.

2. Has the statistical analysis been performed appropriately and rigorously?

Reviewer #1: Yes

Response: Dear Reviewer, we are thankful for the appreciation for are statistical rigor.

3. Have the authors made all data underlying the findings in their manuscript fully available?

The PLOS Data policy requires authors to make all data underlying the findings described in their manuscript fully available without restriction, with rare exception (please refer to the Data Availability Statement in the manuscript PDF file). The data should be provided as part of the manuscript or its supporting information, or deposited to a public repository. For example, in addition to summary statistics, the data points behind means, medians and variance measures should be available. If there are restrictions on publicly sharing data—e.g. participant privacy or use of data from a third party—those must be specified.

Reviewer #1: Yes

Response: Dear Reviewer thanks for your appreciation

---

4. Is the manuscript presented in an intelligible fashion and written in standard English?

PLOS ONE does not copyedit accepted manuscripts, so the language in submitted articles must be clear, correct, and unambiguous. Any typographical or grammatical errors should be corrected at revision, so please note any specific errors here.

Reviewer #1: Yes

Response: We thank the reviewer for this positive assessment. We have carefully proofread the revised version to correct any remaining typographical and grammatical errors.

5. Review Comments to the Author

Reviewer #1 Comment:
The article is well written by the authors.

Response:
Dear Reviewer thank you for the positive evaluation of our manuscript.

Reviewer #1 Comment:
The abstract needs to be modified.

Response:
Dear Reviewer, Thank you for this suggestion. The abstract has been revised to improve clarity and to better reflect the objectives, methodology, key results, and conclusions of the study.

Reviewer #1 Comment:
A flow diagram or schematic diagram of the work may be included.

Response:
Dear Reviewer, we thank you for the suggestion. A stepwise schematic diagram illustrating the complete workflow of the proposed methodology has been included in the manuscript (**Figure 2**) to enhance clarity and readability.

Reviewer #1 Comment:
The need for LASSO and its significance can be explained.

Response:
Dear Reviewer, Thank you for this valuable comment. We have added a detailed explanation of the motivation for using LASSO, highlighting its role in feature selection, dimensionality reduction, and prevention of overfitting in the revised

manuscript in section **3.4 Comparative Analysis with Individual Feature Extraction Techniques**

.

Reviewer #1 Comment:
Why PCA-MI is chosen for analysis instead of KL Divergence?

Response:
Dear Reviewer, The rationale for selecting PCA-MI over KL Divergence has now been clearly explained in the revised manuscript, emphasizing the components preserved from PCA were ranked further by the MI scores that they achieved, which is an essential step in moving toward further tuning the features toward classification. **(Refer Section 3.3)**

Reviewer #1 Comment:
MCC, Kappa, and Dice coefficient may be included.

Response:
Dear Reviewer, we appreciate this suggestion. The Matthews Correlation Coefficient (MCC), Cohen's Kappa, and Dice coefficient have now been included as additional performance evaluation metrics to provide a more comprehensive assessment of the model. **(Refer section 4.4)**

Reviewer #1 Comment:
The conclusion may be modified.

Response:
Dear Reviewer, Thank you for this comment. The Conclusion section has been revised to more clearly summarize the main findings, highlight the significance of the proposed approach, and outline potential future research directions.
